# Spectral Fingerprinting of Engineered Nanomaterials for Precision Biosensing

**DOI:** 10.1021/acsnano.5c18348

**Published:** 2026-01-26

**Authors:** Aceer Nadeem, Maryam Rahmani, Yibo Wang, Sepehr Yari, Rodrigo Monroy Lopez, Mijin Kim, Daniel Roxbury

**Affiliations:** 1 School of Chemistry and Biochemistry, 1372 Georgia Institute of Technology, Atlanta, Georgia 30332, United States; 2 Department of Chemical, Biomolecular, and Materials Engineering, 4260 University of Rhode Island, Kingston, Rhode Island 02881, United States

**Keywords:** optical spectroscopy, engineered nanomaterials, machine learning, feature engineering, optical sensors

## Abstract

Biological systems comprise a complex milieu of macromolecules, small molecules, and ions comprising tens of thousands of distinct species. Various clinical conditions alter the identities and concentrations of these species in a spatiotemporal-dependent manner. While bioanalytical methods such as omics or biochemical assays can precisely identify the targeted biomolecules over space and time, providing in-depth information on biological processes, they are generally considered low-throughput and costly. Spectral fingerprinting of engineered nanomaterials (SFEN) has emerged as an alternative method that addresses many of these limitations in the field of disease detection and chemical biology research. This approach leverages one or more closely related types of engineered nanomaterials to detect subtle biological differences via optical readout such as near-infrared fluorescence or surface-enhanced Raman spectroscopy. Variations of the technique have been developed to detect single or multiplexed target biomarkers as well as whole-cell- and organism-level biological states. In recent years, the incorporation of advanced analytical methods, such as feature extraction and machine learning, has significantly expanded the SFEN capabilities for broader applications with high accuracy. This perspective highlights recent developments of SFEN applications including but not limited to machine-learning-assisted live-cell phenotyping, serum-based cancer detection, and pathogen identification. We further comment on the future directions of this promising technology, which we envision will synergize with next-generation nano-omics and generative AI methods.

## Graphical abstract

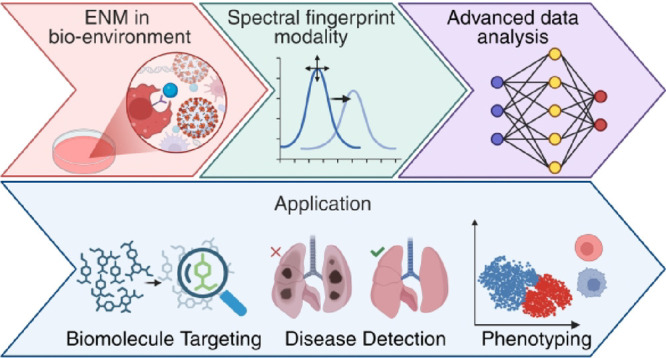

## 1 Introduction

Traditional biosensing approaches have significant challenges in sensitivity and specificity in diagnosing complex diseases. Recently, it has been shown that a few established biomarkers, e.g., cancer antigen 125 and human epididymis protein 4 for ovarian cancers, when measured in the blood, still fail to detect early stage disease states, resulting in poor survival rates.^1^ While mass spectrometry-based diagnostic methods, such as proteomics and transcriptomics, provide rich information on biomarker levels and can yield disease signatures for diagnosis, they require complex facilities and are time-consuming, costly, and not easily scalable.^2^ Similarly, strategies for microbiological diagnosis of bacteria rely on phenotypic characterization through cultivation on chromogenic media, often combined with DNA detection methods such as polymerase chain reaction (PCR) or multiomics data. These conventional techniques typically require hours to days to diagnose a condition.^3^ An alternative biosensing method that is accurate, cost-effective, fast, and scalable is therefore essential.

Spectral fingerprinting, i.e., the technique of identifying unique signatures in spectroscopic data, uses multiple sensors^4^ or multireadout sensors to record complex signals, creating a composite signature of a sample rather than measuring a few targeted molecular interactions^1^ and yielding a single output. Each molecular species has a unique set of vibrational^5^ or electronic^6^ transitions, giving rise to distinct spectroscopic signatures. The specific molecular structure, subtle molecular interactions, and local environment influence the exact frequencies or wavelengths at which a substance absorbs, emits, or scatters light. These spectral patterns serve as identifiable “fingerprints” for different molecules or binding events, enabling precise identification or classification in characterization methods such as mass spectroscopy,^7,8^ Raman spectroscopy,^5,9^ and Fourier-transform infrared spectroscopy (FTIR).^10^ The approach has been applied across diverse fields for the detection of organic contaminants,^11^ the classification of microplastics,^12^ and the source differentiation of ENMs,^13^ collectively representing a new paradigm in biosensing. SFEN can address disease heterogeneity^14^ and capture subtle variations in signatures capable of discriminating biomolecules.^2,9,15,16^ This technology has the potential for large-scale screening due to its high accuracy, cost-effectiveness, simplicity, and rapid detection protocol.^17^

Nanomaterials, i.e. those that have at least one dimension in a range of 1–100 nm, exhibit unique optical, electrical, and chemical properties.^18,19^ Various types of engineered nanomaterials (ENMs) have been synthesized by either top-down or bottom-up methods^20,21^ with assorted dimensions, shapes, surface charges, and physicochemical properties. Such features make them suitable for use in biological applications, including biosensing,^20–23^ drug delivery,^18^ and bioimaging.^24^ ENMs can combine multiple functional entities within a single particle,^24,25^ which facilitates greater sensitivities in in vitro, ex vivo, and in vivo diagnostics.^26^ Functionalization of ENMs can be accomplished with ions, small molecules, and macromolecules such as proteins and other biopolymers, to enhance their biocompatibility and functionality.^27^ Additionally, when nanoparticles enter biological environments, they can adsorb proteins onto their surfaces, forming a dynamic layer known as the biomolecular corona. This corona can alter the physical, chemical, and biological characteristics of nanoparticles and their sensing performance.^15^ However, conventional ENM-based biosensors still face critical limitations in disease diagnostics. Most of them are designed to target one biomarker and produce a low-dimensional signal (e.g., a single fluorescence intensity or color change). In practice, diseases often involve multiple biomarkers produced from complex biological processes, so sensing strategies confined to one signal channel are insufficient to capture subtle, system-wide environmental changes in dynamic molecular profiles and lack comprehensive insight.^28^ The synergy of nanomaterials and the spectral fingerprinting enables amplification of optical signals in spectroscopy techniques (e.g., Raman Spectroscopy^29^) and enhances specificity and multiplexing,^30^ addressing the challenges of conventional ENM-based biosensors in disease detection.

We introduce here the concept of spectral fingerprinting of engineered nanomaterials (SFEN), a sensing approach that employs the unique optical spectra produced by nanomaterials to identify and detect target bioanalytes (Figure 1). SFEN can produce or improve detectable signals with higher stability,^31^ sensitivity, selectivity,^32^ and capacity for multiplexing^33^ of different targets or disease states by their distinct spectral fingerprints.^3,29^ This represents a significant advancement over traditional ENM sensors, which typically require separate labeled probes or channels for each analyte. The spectrum is influenced by the electron distribution states, surface features, and the behavior under mechanical forces of the target molecule or molecules.^29^ For example, adsorption of analytes onto functionalized single-walled carbon nanotubes induces solvatochromic shifts and photoluminescence modulation, driven by changes in the local dielectric environment and electronic interactions at the nanotube surface.^34^ Surface-enhanced Raman spectroscopy (SERS)^17,30,32^ and fluorescence spectroscopy^2,35^ are the most common SFEN methods. Various classes and subclasses of ENMs, e.g., metal oxide,^36–38^ magnetic,^29,37,39^ and bimetallic^32,37^ nanoparticles and quantum dots,^40^ enable high sensitivity in SERS through plasmonic hot spots and analyte surface charge transfer.^9,36^ Also, ENMs, e.g., semiconducting single-walled carbon nanotubes (SWCNTs),^41,1,3,42,43^ and polymer nanoparticles^44^ have been utilized in fluorescence-based SFEN. This method relies on adsorption-induced modulations in local dielectric environments.^45,46^

**Figure 1:**
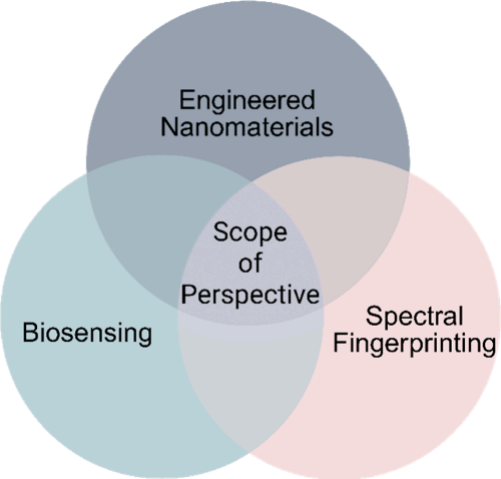
Scope of the perspective positioned at the intersection of engineered nanomaterials, biosensing, and spectral fingerprinting.

SFEN signals comprise high-dimensional spectral features that are often convoluted with overlapping biological background signals and are thus too complex for conventional statistical analysis.^1^ Advanced analytical tools such as machine learning (ML) can assist in detecting subtle differences and pattern recognition,^2^ identifying the biological target(s), or discriminating between different conditions.^2,3,31,38,47^ Researchers have achieved powerful results by implementing ML to SFEN methods, for instance, discriminating proinflammatory from prohealing phenotypes of cultured macrophages that would otherwise appear identical.^41^

This perspective highlights recent advancements in SFEN and their applications in next-generation biosensing and diagnostics (Figure 2). We begin with the optical modalities of spectral fingerprinting, including fluorescence and SERS, and describe the major classes of ENMs used in these methods. We distinguish recent SFEN approaches into in vitro, ex vivo, and in vivo classifications. Advanced analytical methods coupled with this technique, including ML for classification and predictive modeling, as well as other multivariate statistics for feature extraction and analysis, are reviewed. Finally, we consider future directions, challenges, and opportunities in this field.

**Figure 2:**
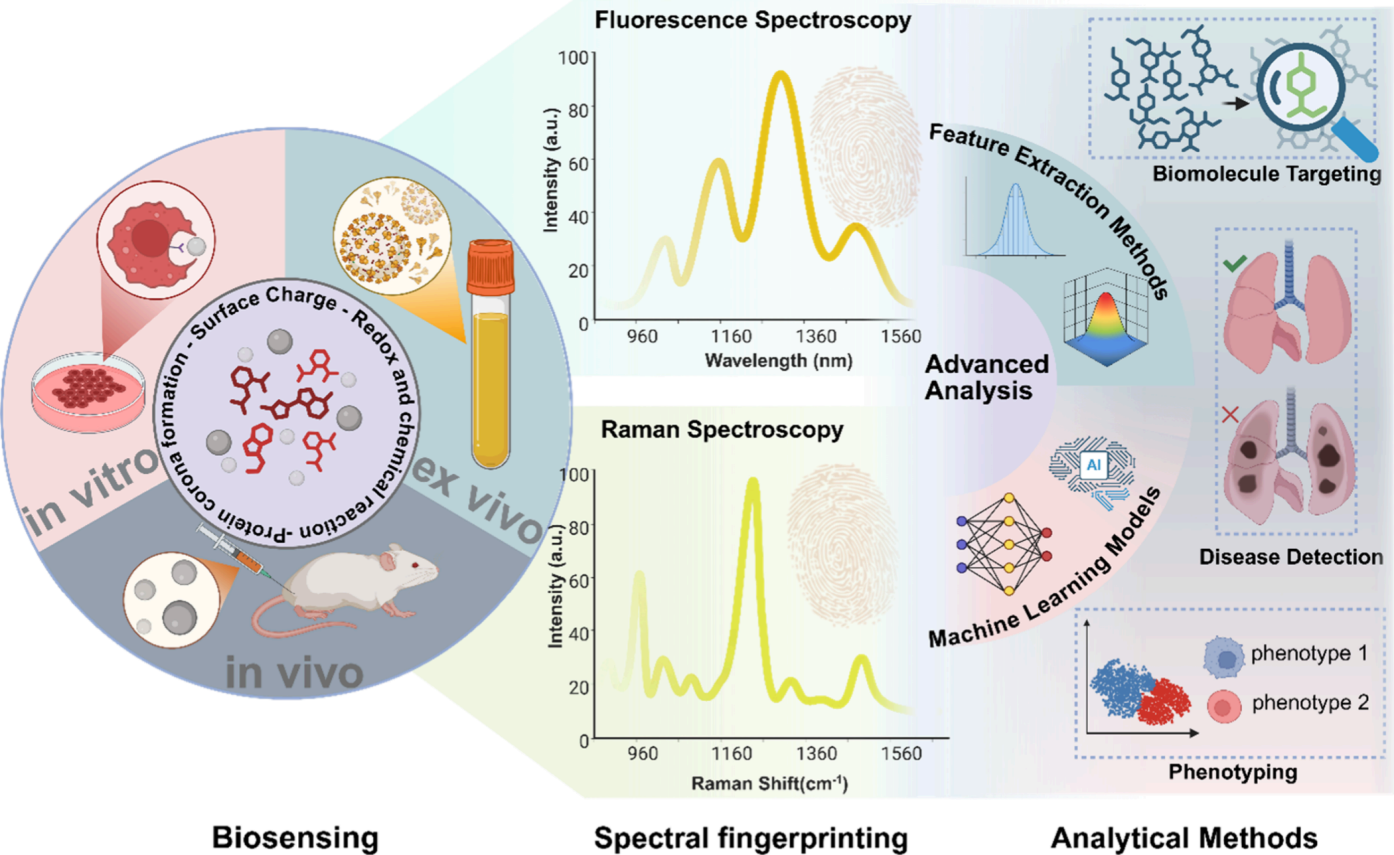
Overview schematic of spectral fingerprinting-based biosensing across in vitro, ex vivo, and in vivo contexts.

## 2 Spectroscopy Modalities and Types of Nanomaterials Used

### 2.1 Surface-Enhanced Raman Scattering

Raman spectroscopy measures the inelastic scattering of photons as they interact with the vibrational modes inherent in the chemical bonds of molecules. It offers high photostability, molecular specificity, and insensitivity to water interference.^9,16,31,37^ However, Raman signals are weak as only a small fraction of photons scatters inelastically; thus, the measurements require a high-intensity power source, resonant excitation, long exposure, and/or signal amplification.^9,16,36^ SERS enhances Raman signals by 3 to 16 orders of magnitude using plasmonic nanostructures.^9,37,48–50^

This enhancement occurs through electromagnetic and chemical enhancement mechanisms (Figure 3a). Excitation of plasmonic metal nanostructure induces collective electron oscillations that concentrate the electromagnetic field into nanoscale “hot spots”. This localized surface plasmon resonances (LSPRs) lead to a strong amplification of both the incoming and scattered Raman fields, roughly following an E4 dependence.^9,36,51,52^ Gold and silver nanoparticles (AuNPs and AgNPs, respectively) are the most common SERS platforms because of their visible spectrum surface plasmon resonance.^53,54^ Their LSPR bands can be tuned by adjusting the particle size, aspect ratio, and spacing.^9,29^ These properties have been exploited in the development of label-free AuNP-based SERS sensors that utilize electromagnetic enhancement to directly fingerprint SARS-CoV-2 RNA, achieving a detection limit of 110 pM, which is within the clinical range of salivary SARS-CoV-2 RNA concentrations.^55^ In another study, investigators reported antibody-conjugated AuNP SERS probes for ultrasensitive identification of mosquito-borne viruses (DENV-2 and WNV), where strong plasmonic coupling within AuNP–virus nanoassemblies enabled detection down to ∼10 PFU/ml.^49^ Furthermore, AgNP-based SERS sensors have been developed for the amyloid-β42 peptide, an Alzheimer’s disease biomarker, enabling discrimination between pathogenic Aβ aggregates and monomeric peptide forms.^56^ This AgNP-based sensor reached an ultralow detection limit of ∼15 pM for amyloid-β42 aggregates in biofluid samples. In the cases where LSPR is not perfectly resonant with the excitation wavelength or for strongly chemisorbed analytes, chemical enhancement plays an important role in SERS. When a molecule adsorbs onto a metal surface, charge transfer between the analyte and the surface may occur either from the highest occupied molecular orbital (HOMO) to the metal Fermi level or from the metal Fermi level to the lowest unoccupied molecular orbital (LUMO), depending on the excitation energy (Figure 3b). In semiconducting ENMs, analogous charge transfer occurs between molecular orbitals of target analyte and the valence or conduction bands of ENMs. These charge transfer processes modify the molecular polarizability and selectively amplify specific vibrational modes.^52,57^

**Figure 3:**
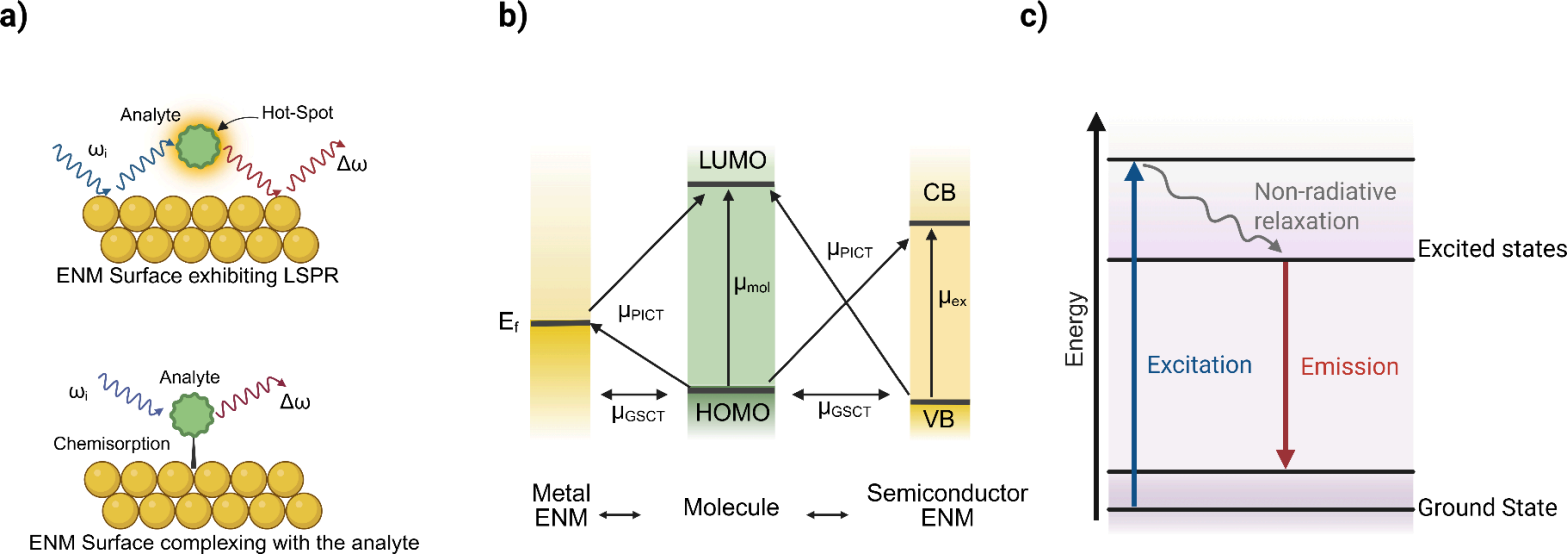
Schematic overview of spectral fingerprinting mechanisms in ENMs. (a) Mechanisms for SERS enhancement include electromagnetic enhancement when an analyte is in the “hot spot” near an ENM (top) and chemical enhancement when charge transfer occurs via a chemisorbed analyte (bottom). (b) Charge transfer between ENMs and HOMO–LUMO states of analytes, including interfacial ground-state CT (μ_GSCT_), photoinduced CT resonance (μ_PICT_), molecular electronic excitation resonance (μ_mol_), and semiconductor excitation resonance (μ_ex_), modulates chemical enhancement of the SERS signal. (c) Generalized mechanism of fluorescence in ENMs.

For signal amplification optimization and hot spot formation, the noble metal substrates (e.g., Ag and Au) are engineered into various shapes like nanospheres, nanorods, nanostars, nanoshells, and nanogaps.^38^ Recent work on composite SERS substrates contains zero-dimensional (0-D) nanomaterials (e.g., quantum dots and carbon dots), 1-D (carbon nanotubes), and 2-D nanomaterials (graphene and MoS_2_), combined with metals to increase both electromagnetic and chemical enhancement. Magnetic nanohybrids, such as iron oxide-based core–shell structures (e.g., Fe_3_O_4_@Ag), facilitate binding of target analytes.^39^ Moreover, dielectric nanostructures, made from high-refractive-index materials like TiO_2_ or ZnO, provide lower-loss enhancement, which prevents heating and allows for more precise control over the spectrum.^9^ SERS does not require long preparation steps that are inherent to conventional biochemical assays.^32,39^ SERS offers both label-free and label-containing detection as well as promoting noninvasive, real-time biosensing.^32,48^

More importantly, SERS is highly sensitive down to attomolar levels,^32^ which is comparable to digital enzyme-linked immunosorbent assay (ELISA) methods.^58^ This sensitivity, along with highly specific vibrational signatures that reflect the molecular structure and chemical environment of analytes and sharp spectral peaks (∼0.1 nm) compared to colorimetry or fluorimetry techniques (∼100 nm),^59^ enables SERS to generate rich information for SFEN in diverse biological systems. When combined with advanced analytical techniques, SERS-based biosensing becomes more powerful by quickly sorting through spectra, identifying patterns, and further increasing accuracy, sensitivity, and specificity of the SFEN approach.^9,16,32^

SERS-based SFEN approaches have been widely applied in various biological environments, e.g. whole blood,^16^ serum,^39^ tissue,^36^ sputum,^9^ and single-cell contexts,^9^ to detect a variety of target analytes including bacterial species,^16^ fungal pathogens,^39^ cancer biomarkers relevant to esophageal, lung, and prostate cancers,^32,36^ and single-cell molecular signatures (e.g., DNA/RNA ratio shifts).^9^

However, certain challenges remain in nanoparticle aggregation, biosafety, and batch-to-batch variation, as discussed in Section 5.2 and Table 1.^32,38,51^ Additionally, tissue penetration is limited (usually under 5 mm),^51^ and in label-free detection, the spectra can be overly convoluted and difficult to interpret.

**Table 1:** Pros and Cons of Different Modality of Spectral Fingerprinting Technique

**modality**	**nanomaterials used**	**pros**	**cons**	**ref**
SERS	noble metal nanostructures (Au, Ag, and Cu: nanospheres, nanorods, nanostars, and nanogaps)	high sensitivity (single cell and molecule)	reproducibility issues (nanoparticle aggregation and batch variability)	^9,16,32,38,50,51,69^
		detection limits down to attomolar ranges	Limited tissue penetration (<5 mm) restricts in vivo use.	
	magnetic hybrids (e.g., Fe_3_O_4_@Ag)	Sharp, narrow peak widths (typically 0.1 nm) enable multiplexed sensing.	Spectral overlaps in complex biological samples make interpretation difficult.	
	composites with CNTs, graphene, and MoS_2_	photostability	requires precise substrate engineering for reproducibility	
	dielectric nanostructures (TiO_2_ and ZnO)	no photobleaching	clinical translation limited by biosafety and scalable synthesis challenges	
		water insensitivity		
		compatible with both label-free and labeled biosensing		
fluorescence	semiconducting SWCNTs	allows deep tissue imaging (several cm) with minimal scattering and autofluorescence	Signal depends strongly on nanotube chirality and bundling, complicating interpretation.	^3,64,63,42,65^
		high sensitivity (pico-nanomolar) and real-time detection	Sample aggregation and serum protein background can reduce signal accuracy.	
		environmental sensitivity to diverse physicochemical interactions e.g., pH, redox, and dielectric changes	Preparation can be labor-intensive (chirality sorting and OCC synthesis).	
	semiconducting polymer nanoparticles (SPNs)	photostability suitable for long-term imaging	Selectivity can be broad (e.g., detecting overall oxidative stress rather than specific species)	
		Biocompatible surface coatings (DNA, PEG, and aptamers) improve stability and targeting.	wider peaks compared to SERS (20–80 nm)	
FTIR/Mid-IR	antibody-functionalized Fe_3_O_4_ magnetic nanoparticles	label-free detection	sensitivity lower than SERS (requires higher analyte concentrations)	^68^
		pathogen detection in <30 min with portable device	susceptible to interference from water and complex biological matrices	
		detection limit of 10^4^–10^5^ CFU/mL in complex food matrices		

### 2.2 Fluorescence Spectroscopy

As shown in Figure 3c, fluorescence in ENMs starts with photon absorption, which creates an exciton that relaxes and then emits light. These excitons are very sensitive to changes in the local dielectric or chemical environment, such as charge transfer or redox activity. Physicochemical interactions between ENMs and adsorbed molecules shift the emission wavelength, change the intensity, and even quench the signal. These environment-dependent optical changes are essentially what generate the spectral fingerprints. Leveraging the aforementioned desired optical properties, as well as high environmental sensitivity, compatibility with complex biological environment, and the ability to provide real-time detection, fluorescence-based methods are widely used in biosensing and SFEN.^1–3^ In addition, the second near-infrared window (NIR-II, 1000–1700 nm) has minimal tissue scattering and autofluorescence. The NIR-II fluorescent nanoparticles, e.g., SWCNTs, thus enable optical signals to travel deeper into tissue (up to several centimeters) with a minimal background interference.^44,60–62^

Fluorescent ENMs, including semiconducting SWCNTs and semiconducting polymeric nanoparticles (SPNs),^1,2,63^ translate biochemical interactions into distinct optical fluorescence patterns (i.e., SFEN). For example, functionalized SWCNTs can sensitively respond to local environmental changes, including pH, redox, and dielectric conditions, and transduce the physicochemical changes into optical modulations that can be interpreted.^64,65^ SWCNTs exhibit excitonic fluorescence, which can be polarized by the local dielectric environment (i.e., solvatochromism) near the surface of the SWCNT. This polarized exciton gives rise to shifts in emission wavelength and therefore enhances the sensing abilities of the SWCNT. The resultant signals are then classified using advanced analytical techniques to detect and quantify specific target molecules.^1,3,43^

Despite their demonstrated advantages, traditional fluorescence-based SFEN for biosensing can be limited by sample aggregation or matrix complexity (for example, nonspecific binding of high-abundance proteins), photobleaching over time, spectral drift, and quenching effects. In contrast, SWCNTs are photostable, can be multiplexed using structure-defined narrow emission bands, and expand chemical diversity via surface functionalization chemistry, thus addressing challenges in traditional fluorescence-based ENMs,^65,66^ including low photostability, limited multiplexing capability, signal drifts, and quenching in biological systems. Nevertheless, to transduce robust signal transduction to target biological states and/or analytes, precise control of physicochemical properties of ENMs is required, for instance, chirality-pure SWCNT sorting, noncovalent wrapping of synthetic polymers, incorporation of quantum defects, or bioconjugations (Table 1).^1–3,43,63,67^

### 2.3 Fourier-Transform Infrared Spectroscopy

Other, less utilized modalities of SFEN exist and have been used in biosensing. FTIR spectroscopy is a label-free optical technique that detects various molecular vibrations, providing complementary information that SERS offers. In biological sensing, FTIR produces spectral fingerprints based on the absorption of mid-IR light by the chemical bonds of biomolecules, such as proteins, lipids, and nucleic acids. These fingerprints provide pathogen identification relying on their biochemical composition, which enables selective and rapid biosensing.^68^

However, FTIR-based approaches generally lack sensitivity due to interference in complex biological environments. While FTIR can be compatible with portable spectrometers, these generally have lower spectral resolution than benchtop instruments (Table 1).^68^

## 3 Biological Contexts and Targeted Analytes

The ability to accurately probe and interpret complex biological processes at the molecular level is fundamental to advancing biomedical research and clinical diagnostics. Conventional analytical methods often face limitations in sensitivity or the capacity to capture the holistic state of a biological system. SFEN translates multifaceted molecular interactions into high-dimensional optical signatures that encode the underlying physiochemical environment of nanobiointerfaces. Unlike conventional nanosensor readouts that rely on single intensity or band-ratio measurements, SFEN leverages the full spectral manifold, capturing shifts, intensity modulations, bandwidth changes, and exitonic coupling to generate mechanistically meaningful fingerprints interpretable through machine-learning algorithms. This multidimensionality enables deeper insights into biological processes and their spatiotemporal transitions (Table 2).

**Table 2:** SFEN-Based Bioanalyte Detection across Sample Types and Targets

**sample type**	**SFEN**	**target**	**detection method**	**data processing**	**performance**	**ref**
in vitro						
macrophage	DNA -SWCNTs	macrophage phenotypes	NIR fluorescence	SVM	>95% accuracy	^41^
primary endothelial cells	DNA -SWCNTs	intracellular endosomal pathway	NIR fluorescence and Raman imaging	ANN	>84% accuracy	^42^
		Endosome type				
HeLa cells	AgNPs	digestion behavior of lysosomes	SERS	structural similarity algorithm	structural similarity index> 0.7 (high correlation)	^78^
Bacteria	polymer (ssDNA, genomic calf thymus DNA, hemin-binding DNA aptamer, LPS-binding peptide conjugated to ssDNA, PEG–phospholipid (DSPE–PEG_5000_), and bovine serum albumin (BSA)) wrapped SWCNTs	pathogen-released virulence factors, including LPS, siderophores, extracellular proteases, and nucleases	NIR fluorescence	PCA for separation; LDA classifies *S. aureus* vs *Staphylococcus epidermidis*	linear discriminant analysis (LDA) posterior ≈ 0.80	^3^
Virus	PEG-lipid–wrapped SWCNT	SARS-CoV-2	NIR fluorescence	CNN-based pattern recognition on full spectra, followed by Shapley additive explanations	99.68% accuracy; LOD (10 ng/mL for V1, 2.5 ng/mL for V2, and 3 ng/mL for V3)	^80^
		MERS-CoV				
		SARS-CoV-1				
Pathogen	Ag-nanoparticle–loaded paper chips paired with 4-mercaptophenylboronic acid (4-MPBA)	10 different pathogens	SERS	multibranch adaptive attention convolutional neural network	98.6% overall species-level accuracy	^81^
					99.5% accuracy between antibiotic-resistant and sensitive	
ex vivo						
serum	DNA-wrapped covalently functionalized SWCNT array	high-grade serous ovarian carcinoma	NIR fluorescence	supervised machine-learning models (SVM and RF) for binary classification	87% sensitivity at 98% specificity	^1^
serum	label-free silver nanoparticle	cardiovascular disease biomarkers, including cTnI, BNP, CRP, LDL, and creatinine	SERS	PCA-reduced linear SVM	test accuracy of 90.0%	^147^
serum exosomes	potassium-iodide-modified silver-nanoparticle film	stage	SERS	random forest (RF) combined with LASSO for feature selection	100% for stage I lung adenocarcinoma	^83^
		subtyping of lung cancers			81% for preneoplasia	
tissue biopsy	silver nanoparticle	Breast cancer	SERS	classification by PCA-LDA, PLS-DA, and SVM	sensitivity of 94.74%	^82^
		Fibroadenoma			specificity of 83.33% for breast cancers	
		Breast hyperplasia				
urine	surface-carbonized Ag nanowire	pancreatic cancer	SERS	classification based on PCA and orthogonal least-squares discriminant analysis (OPLS-DA)	pancreatic cancer: sensitivity, 95%; specificity, 92.9%; prostate cancer: sensitivity, 97.2%; specificity, 94.4%	^17^
		prostate cancer				
serum	Fe_3_O_4_@PEI magnetic NPs and silver nanoparticle	*C. albicans*, *Candida tropicalis*, and *Candida krusei*	SERS	OPLS-DA using species-characteristic fingerprint bands	99.8% test accuracy	^39^
in vivo						
plant	polymer-wrapped gold nanosphere	stress signaling molecules, including salicylic acid, ATP, cruciferous phytoalexin, and glutathione	SERS	multiplex detection by distinct Raman bands and Langmuir binding model for quantification	early diagnosis: SA/eATP detectable ∼2 h (day 0) post fungal inoculation, preceding visible lesions/PCR	^91^
plant	DNA-SWCNT, cationic fluorene–diazine copolymer S3–SWCNT	H_2_O_2_, salicylic acid	NIR fluorescence	SA concentration quantification by Langmuir calibration	SA sensor: LOD = 4.4 μM	^90^
mouse	QDs (AuNP@Raman dye–silica shell)	solid tumor	NIR-SERS	nonnegative Levenberg–Marquardt–Fletcher (LMF) least-squares algorithm	LOD: 3.8 ± 0.38 fM (red), 21.2 ± 6.6 fM (blue)	^94^
mouse	DNA-SWCNTs	doxorubicin	NIR fluorescence	monitor wavelength shift and intensity change through nanotube-loaded membrane-based implant device	sensitivity: 50 μM in vivo	^92^

### 3.1 In Vitro Models and Biosensing Strategies

In vitro biological environments, ranging from 2D cell cultures to isolated microbial samples, serve as the foundational platform for validating SFEN technologies. The primary advantage of SFEN in this context is its ability to interrogate living systems nondestructively and in real time. By leveraging the environmental sensitivity of ENMs, researchers can capture dynamic molecular fingerprinting that reflects the functional state of a cell or pathogen. This contrasts with traditional end point assays, which often require cell fixation or lysis, thereby compromising temporal resolution.

In this section, we categorize in vitro SFEN applications into two primary domains: disease phenotyping, where high-dimensional spectral data are used to decipher complex cell states such as immune polarization and intracellular trafficking; and pathogen detection, where distinct spectral signatures enable the rapid, multiplexed identification of bacteria and viruses.

#### 3.1.1 Disease Phenotyping

Single-cell and subcellular level SFEN methodologies using high-throughput optical and mass spectrometry have the potential to spatially and/or temporally capture heterogeneous biological responses in cell cultures, such as differential cell cycle states, metabolic activity, gene expression, and nanoparticle uptake.^70,71^ At this scale, polymer-wrapped SWCNTs can serve as intracellular reporters since noncovalent functionalization of certain biocompatible polymers on the SWCNT sidewall forms a unique recognition interface for potential target binding.^72,73^ This polymeric corona phase determines the molecular binding affinity and SWCNT emission responsiveness to the local milieu.^72^ When internalized into cells, the polymer SWCNTs respond to local physicochemical cues such as pH,^74^ ionic strength,^75^ enzymatic activity^76^ (e.g., DNase II and APE-1), proteins,^77^ and other biomolecules that are physiosorbed onto the SWCNTs, resulting in distinct changes in their NIR emission profile. For instance, ssDNA-wrapped SWCNTs (DNA-SWCNTs) exhibited distinct fluorescence responses across M1, M2, and naive macrophage phenotypes (Figure 4A).^41^ These spectral fingerprints reflected endolysosomal environments within distinct macrophage phenotypes and protein environments. Mechanistically, macrophage polarization entails complex metabolic shifts; for instance, the M1 phenotype exhibits heightened lysosomal acidity and elevated reactive oxygen species (ROS). These physiological factors collectively modulate the local dielectric constant and electronic bandgap states of the SWCNTs, inducing distinct wavelength shifts and peak broadening. Unlike conventional sensors that typically report a single scalar value for a specific target, SFEN captures this multidimensional response. This enables the resolution of subtle, heterogeneous phenotypic states that would be indistinguishable using single-channel fluorescence readouts. When coupled with machine learning, these multidimensional spectra achieved >95% accuracy in phenotype identification, demonstrating that spectral fingerprinting captures latent correlations between biological states and photophysical responses, far beyond traditional fluorescence fingerprinting.

**Figure 4:**
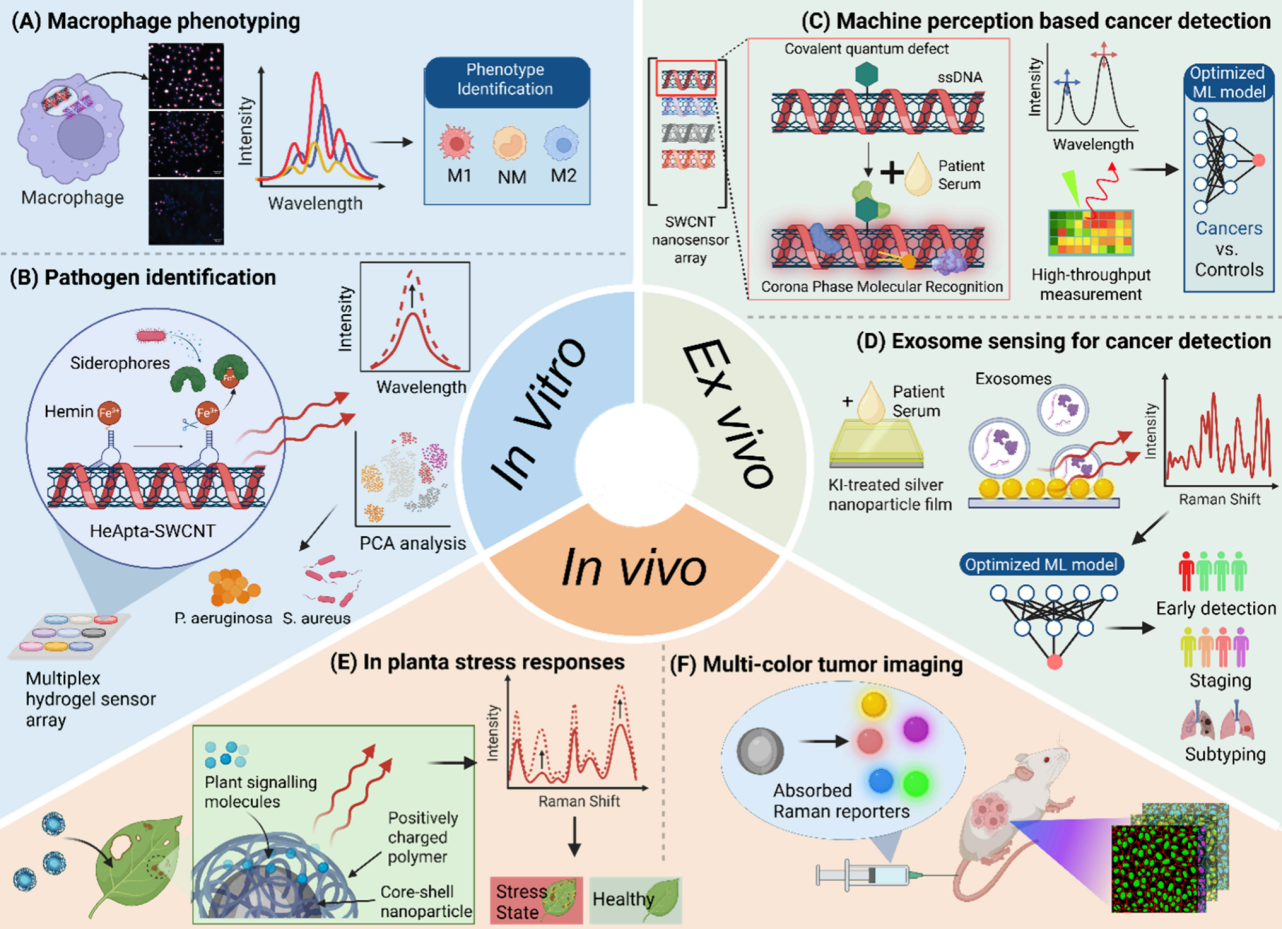
Applications of SFEN across biological contexts. In vitro: (A) Macrophage phenotyping using machine-learning-assisted spectral fingerprint of DNA-SWCNT sensors.^41^ (B) Bacterial identification using polymer-wrapped SWCNT incorporated into a hydrogel sensor array.^3^ Ex vivo: (C) Ovarian cancer detection via the spectral fingerprinting of functionalized carbon nanotubes in serum.^1^ (D) Lung cancer diagnosis by optical deciphering of serum exosomes spectral fingerprints. In vivo: (E) In planta diagnosis of stress states with the Raman enhancement on the surface of PDDA-coated silver nanoparticles.^91^ (F) In vivo multiplexed tumor imaging by five colors of Raman-labeled nanoparticles.^94^

Beyond phenotypic classification, ENMs enable high-resolution interrogation of nanobiointeractions and intracellular trafficking dynamics. For instance, NIR fluorescence and resonance Raman scattering-based SFENs can monitor the intracellular activity of DNA-SWCNTs in primary human endothelial cells.^42^ Longitudinal monitoring of the multispectral features revealed that dynamic dielectric and charge transfer effects as DNA-SWCNTs were trafficked through early and late endosomes. Time-dependent redshifts, intensity reduction, and broadening in emission bands, along with increased G-band intensity in Raman, were observed. The spectral changes indicated that DNA-SWCNTs underwent aggregation within cells over time, particularly in late endosomes and lysosomes, where increases in acidity, protein content, and SWCNT concentration have been observed. DNA-SWCNTs also exhibited organelle-specific spectral patterns corresponding to early and late endosomes, as confirmed by immunofluorescence colocalization. These spectral changes correlated with luminal acidification, protein concentration, and vesicle maturation stages, highlighting the ability of SFENs to resolve spatiotemporal variations in intracellular microenvironments.

Moreover, other ENMs like gold nanoparticles have been used to generate distinct spectral fingerprints to decode the macromolecular digestion behavior within lysosomes of live single HeLa cells.^78^ These fingerprints reflected the local degradation of macromolecules, such as proteolysis, nucleic acid cleavage, and lipid breakdown. This information was selectively extracted by an innovative method that leveraged the structural similarity algorithm to correlate hyperspectral SERS images with lysosomal fluorescence images, providing quantitative molecular signatures of metabolic enzyme activity within single cells by identifying key degradation-related Raman bands with high correlation between the fingerprinting spectra of some molecular chemical vibrations and lysosomal metabolism.

#### 3.1.2 Pathogen Detection

Pathogen identification represents a critical area in biosensing due to the medical urgency of rapid and precise diagnostics. It also requires simultaneous sensitivity and specificity under heterogeneous sample conditions. SFEN platforms achieve these through multidimensional, label-free spectral encoding combined with interpretable ML models capable of distinguishing subtle differences in viral or bacterial biomolecular composition.

A hydrogel-based sensor array composed of polymer-wrapped SWCNTs enabled label-free, multiplexed pathogen detection with high sensitivity and specificity (Figure 4B).^3^ The array included a series of noncovalent coatings of SWCNTs using ssDNA, synthetic polymers, and biomolecules, such as PEG-lipids, BSA, and denatured calf thymus DNA. The biomolecular coronas formed on the SWCNT surface conferred differential sensitivity to lipopolysaccharides, iron-chelating siderophores, proteases, nucleases, and general protein or pH changes, mimicking the secretome of clinically relevant pathogens. This SFEN platform demonstrated the ability to differentiate clinically relevant bacterial species, including *Staphylococcus aureus*, *Escherichia coli*, and *Pseudomonas aeruginosa*, by capturing distinct spectral fingerprints associated with lipopolysaccharides, siderophores, and enzymes. This capability highlights the superiority of SFEN over traditional ligand–receptor sensors. Instead of relying on a binary binding event, the sensor array interacts with the pathogen’s entire secretome—a complex mixture of metabolites and proteins. The resulting spectral fingerprint is a composite of multiple optical modulations. By decoding these high-dimensional data via machine learning, SFEN can identify pathogens at the strain level, providing a depth of biological insight into microbial identity that single-analyte sensors cannot achieve.

PEG-lipid-functionalized SWCNT sensors were developed to detect nucleocapsid proteins from coronaviruses, including SARS-CoV-2, MERS-CoV, and SARS-CoV-1.^79^ The PEG lipids were chemically modified to vary the chain length, fatty acid saturation, molecular weight, and end-group moieties. High-throughput automated screening of the SWCNT array demonstrates how the physicochemical properties of the PEG-lipid-SWCNT 3D corona interfaces correlate with the viral detection efficiency. In aid of the machine-learning process to decode hidden features in the NIR fluorescence spectra, the PEG-SWCNT nanosensors achieved multiplexed detection of coronaviruses at ultralow concentrations.^80^ A convolutional neural network (CNN) was employed to extract and integrate global spectral patterns, to decode analyte-specific hidden multispectral features within NIR fluorescence spectra, resulting in sensitive, accurate classification of virus type with limits of detection (10 ng/mL for SARS-CoV-2, 2.5 ng/mL for MERS-CoV, and 3 ng/mL for SARS-CoV-1), which are 80 times below the classically defined limits of detection. With fine-tuning using 80 samples, the prediction model can be applied to human serum samples, maintaining a high accuracy of 92.1%.

Silver-nanoparticle-loaded paper chips enabled culture-free identification of 10 clinical pathogen species using SERS.^81^ When bacterial samples are treated with 4-mercaptophenylboronic acid (4-MPBA), an interaction between the boronic acid and the diol group of the saccharide in the bacterial cell wall forms a cyclic boronate ester. The thiol group of the bacteria-4-MPBA conjugate can be covalently attached to the silver surface. The newly formed chemical structure between 4-MPBA and bacteria can produce a Raman signal with a high spectral diversity. The resulting spectra were processed by a multibranch adaptive attention CNN. Due to the specific bacteria immobilization to the SERS substrate and enhanced spectral diversity, the SERS chip achieved 98.6% overall species-level accuracy and 99.5% accuracy for distinguishing between antibiotic-resistant and sensitive strains.

### 3.2 Ex Vivo Biosensing in Isolated Biological Matrices

Ex vivo SFEN techniques leverage clinically accessible biological specimens, such as blood, saliva, urine, and tissue biopsies, to provide molecular and pathophysiological insights for diagnostic applications. Recent advances in ENMs have enabled their spectral decoding with high sensitivity and specificity to various biomarker types, offering critical insight for early disease detection, monitoring, and stratification.^32,38,39,82,83^

#### 3.2.1 Blood-Based Fluids

Blood samples (e.g., serum and plasma) contain key biomarkers including proteins, metabolites, circulating tumor DNAs, lipids, extracellular vesicles (EVs), and circulating tumor cells (CTCs). SERS-based SFEN of iodide-modified silver nanofilms combined with a CNN and binary classification has enabled label-free profiling of serum exosomes in lung cancer patients, detected stage I adenocarcinoma with 100% accuracy, and further triaged lung cancer subtypes with 81% accuracy (Figure 4D).^83^ Additionally, gold-coated magnetic nanoparticles have been used to enrich and detect EVs via multiplexed Raman reporters, enhancing specificity in complex patient samples.^83^

In liquid biopsies, protein biomarkers remain essential to clinical diagnostics, and again, gold nanoparticles play an important role due to their tunable surface plasmon resonance and bioconjugation flexibility. Gold nanoparticle-based hybrid platforms that integrate SERS with fluorescence have enabled quantitative, multiplexed assays for protein biomarkers such as C-reactive protein, neuron-specific enolase, and interleukin-6 in serum.^84^ In another study, an electrokinetically enhanced quantum defect SWCNT sensor array bioconjugated with antibodies has been developed for multiplexed detection of six ovarian cancer biomarkers, including cancer antigen 125, human epididymis protein 4, matrix metalloproteinase-7, interleukin-6, and hepatocyte growth factor, in serum.^42^ This platform demonstrated a limit of detection in the low picomolar range for each target and achieved classification accuracies exceeding 90% for patient serum samples. Compared to conventional ELISA-based assays, the SWCNT platform offered at least an order of magnitude improvement in sensitivity, along with faster, multiplexed readouts.^1^

Furthermore, the detection of microorganisms, including fungi, has been accomplished with SFEN approaches, combining SERS with magnetic enrichment. Hu et al. developed a label-free platform where antibody-functionalized magnetic nanoparticles selectively capture *Candida albicans* from patient serum samples.^39^ These complexes are then probed using silver-nanoparticle-based SERS enabling identification without additional labeling. Multiple peaks were identified as key spectral features related to *Candida* cell wall components, which allowed for rapid and accurate detection with 99.8% accuracy achieved under 40 min.

Finally, even when no effective target biomarkers exist, SFEN has been shown to detect disease fingerprints via a machine perception approach. SWCNTs were covalently modified by fluorescent quantum defects and noncovalently wrapped by ssDNA to create an array of NIR fluorescent sensors. When exposed to patient serum samples, the functionalized SWCNTs transduced disease-specific spectral responses by a differential biomolecular corona composition on the SWCNT surface. The specific advantage of SFEN lies in its sensitivity to environmental heterogeneity. Ovarian cancer alters the global composition of the serum proteome and not just the levels of a few marker proteins. These dysregulated biomolecules compete for adsorption on the nanotube surface, creating a disease-specific corona phase. Thus, SFEN effectively performs a “pan-omic” analysis of the serum environment, offering superior diagnostic accuracy compared to traditional immunoassays that are limited to detecting single, often nonspecific, biomarkers. This nanosensor array platform, combined with machine learning, achieved over 87% sensitivity at 98% specificity to detect high-grade serous ovarian carcinoma (Figure 4C).^1^

#### 3.2.2 Saliva and Urine

Saliva and urine offer distinct advantages, such as noninvasive collection and patient compliance. Recent efforts in SFEN have leveraged these fluids to uncover diagnostic signals across a broad range of analytes with high sensitivity. In urinary analysis, SERS profiling of EVs using gold nanoparticles functionalized with Raman active dyes specifically 4-mercaptobenzoic (4-MBA) has been shown to capture disease-specific lipid and protein spectral signatures, particularly in urological cancers.^17,38^ Magnetic–plasmonic hybrid nanoparticles were designed to enrich EVs by combining magnetic cores for rapid separation with antibody-functionalized plasmonic surfaces for the specific capture of EV surface proteins. In addition, the hybrid nanoparticles stabilized colloidal interactions in the dilute urinary environment prior to signal analysis.^32^ Beyond vesicles, SWCNT-based platforms sensitive to urea and creatinine have demonstrated real-time, fluorescence-based sensing capabilities, pushing urine analysis toward robust and continuous diagnostic formats.^85^

Saliva is gaining traction in pathogen detection and inflammatory monitoring. Gold nanoparticle-based SERS platforms have been shown to detect *Streptococcus* mutants from unprocessed saliva, revealing pathogen-specific spectral features.^84^ Saliva has also been explored for viral fingerprinting using silver-nanoparticle-enhanced SERS to detect respiratory infections.^37^ Complementing these, SERS devices have been adapted for multiplexed cytokine detection in saliva for real-time immune profiling.^32^ While the presence of CTCs in saliva or urine is less consistent than in blood, different strategies, such as nanoparticle-based epithelial cell enrichment, are beginning to translate SFEN tools into these matrices.^86,87^

#### 3.2.3 Biopsy Tissues and Fine Needle Aspiration Samples

Biopsy tissues remain clinical mainstays for cancer diagnosis and molecular characterization, offering direct access to biomolecular composition in solid tumors. Gold and silver-nanoparticle-enabled SERS-based SFENs have been widely adopted in fixed and fresh biopsy specimens.^88^ For instance, gold nanostars functionalized with distinct Raman reporter molecules such as 4-MBA and *p*-aminothiophenol (PATP) were applied to breast cancer tissue biopsies.^32^ These gold nanostars with multiplexed Raman reporters simultaneously map proteins and metabolic changes in tissue histology with high spectral fidelity, offering insights into tumor heterogeneity that is often inaccessible via traditional immunohistochemistry.

Fine needle aspirates, often limited in volume and cellularity, benefit from the high signal amplification provided by nanomaterial-based SERS. In one study, cancerous cells extracted from fine needle aspirate samples were incubated with gold-silica core–shell nanoparticles to generate unique Raman signatures reflective of malignancy. This allowed rapid classification of samples without cytological staining, potentially accelerating decision making in clinical environments.^82^ Moreover, quantum defect-functionalized SWCNTs have been explored for the detection of glioma, leukemia, and glioblastoma via metabolic shifts induced by the production of oncometabolites and the accumulation of enzyme–substrate metabolites within fine needle aspirates, and for tracking tumor-derived biomolecules in situ.^43^

#### 3.2.4 Breath Condensates

Exhaled breath and its condensates are increasingly recognized as rich, noninvasive diagnostic fluids, offering a window into metabolic and infectious processes via the detection of volatile organic compounds (VOCs), proteins, and pathogen-derived biomolecules. Unlike blood or tissue-based matrices, breath analysis presents a unique physicochemical challenge due to its gaseous phase, low analyte concentration, and dynamic composition.^84^ Advances in ENMs and SFEN approaches have enabled the selective capture and optical readout of clinically relevant breath composites, multiplexing potential, rapid readout, and translation into field-deployable applications.

Carbon nanotube-based sensor platforms, including Raman sensors functionalized with ionic liquids (IL), have demonstrated selective detection of VOCs in exhaled breath relevant to pulmonary diseases. In this system, SWCNTs were noncovalently blended with imidazolium-based ILs to create sensor arrays capable of discriminating between aldehydes and aromatic compounds associated with diseases such as tuberculosis and lung cancer. The selective response arises from tailored interactions between the IL functional groups and specific VOCs, enabling the reproducible and reversible detection of biomarkers in exhaled breath condensates. Principal component analysis (PCA) of sensor responses revealed clear discrimination of disease relevant VOC profiles under both dry and humid conditions, supporting the potential for noninvasive respiratory disease diagnosis.^89^

### 3.3 In Vivo Spectral Fingerprinting of Biological Systems

Plant health monitoring is central to global food security, driving demand for nondestructive, real-time, in planta sensing that can detect biochemical changes prior to the onset of visible symptoms. SFEN-based approaches in plant systems enable real-time, noninvasive monitoring of physiological responses, nanomaterial interactions, and stress signaling processes. By modulating surface charge through the noncovalent wrapping of synthetic polymers, SWCNTs can efficiently translocate into plant cellular compartments such as chloroplasts and cytoplasm. A recent study demonstrated the multiplexed detection of stress-related signaling molecules by codelivering cationic polymer-wrapped SWCNTs and DNA-SWCNTs into the same leaf.^90^ This approach enabled the observation of distinct temporal patterns in the local generation of salicylic acid with an LOD of 4.4 μM. SERS-active ENMs, consisting of silica nanospheres encapsulated by corrugated silver shells and functionalized with a cationic polymer, poly­(diallyl dimethylammonium chloride), were developed to nondestructively detect multiple endogenous stress-related molecules in living plants (Figure 4E).^91^ The ENMs were introduced into the apoplast via stomatal infiltration and exhibited strong Raman fingerprints to endogenous stress messengers at the micromolar level including salicylic acid, extracellular ATP, glutathione, and phytoalexin-like metabolites. Quantitative monitoring showed that salicylic acid and eATP were detectable approximately 2 h after fungal inoculation, a time frame that precedes both visible lesions and positive PCR results. The study achieved multiplexed detection of abiotic and biotic stresses of plants, providing crucial insights into early stage stress management in plants.

Though demonstrated applications in animal models remain nascent, SFEN has shown emerging promise in in vivo animal models by enabling multiplex, noninvasive detection of biochemical processes underlying disease progression and therapeutic response. Compared to traditional fluorescence tracking or MRI contrast imaging, where each probe typically reports a single analyte and suffers from scattering or photobleaching, SWCNT- and quantum dot-based SFEN probes exhibit narrow near-infrared (NIR-II, 1000–1400 nm) emission bands, enhanced photostability, and minimal background autofluorescence, yielding signal-to-noise ratios (SNRs) exceeding 30–50 under physiological imaging depths. Such optical properties allow spectral deconvolution of multiple analytes in vivo, enabling multiplexed visualization of tumor biomarkers, reactive oxygen species, metabolic intermediates, and biochemical processes like apoptosis or immune activation, offering a window into treatment dynamics.^1,43^ These tools have been successfully deployed to monitor drug responses in live animal models.^92^

In pharmacokinetic studies, PEGylated SWCNTs showed blood circulation half-lives of 18.9–22.1 h. The optical signals remained stable during repeated imaging sessions, allowing temporal mapping of drug-induced apoptosis and immune activation dynamics. This extended circulation time, combined with high photostability, enables longitudinal monitoring of disease progression or treatment response over days, a capability often lacking in rapidly cleared small-molecule dyes.^93^

SERS-enabled SFEN nanostructures extend this performance by combining the deep tissue penetration of NIR light with spectrally distinct Raman fingerprints. For instance, NIR-SERS gold multicore–silica nanoparticles have also been used to achieve noninvasive five-color Raman imaging of tumors in living mice (Figure 4F).^94^ Remarkably, effective imaging was achieved at nanoparticle doses in the femtomolar range, with longitudinal monitoring sustained for up to 24 h, demonstrating high sensitivity and multiplexing capability for in vivo tumor diagnostics. Quantitatively, this method achieved a limit of detection as low as ∼3.8 fM in vivo, which is orders of magnitude more sensitive than typical fluorescence contrast agents. Although these SERS readouts are exogenous reporter fingerprints rather than endogenous disease signatures, multichannel spectral fingerprints can be reliably recovered in vivo, foreshadowing SFEN-based multiplex diagnostics.

## 4 Advanced Analytical Approaches

### 4.1 Data Processing and Feature Engineering

Rigorous data preprocessing and feature engineering are critical for robust, high-performance outcomes in SFEN approaches. Raw spectroscopic data are susceptible to signal variations caused by instrumental noise, background contamination, environmental fluctuations, and sample variability, which can obscure true analytical signals.^95,96^ Deconvolution of target signals is another major challenge due to the complex spectral responses of ENMs. SFEN spectra often exhibit overlapping features, and when multiplexed, distinguishing specific target responses becomes even more challenging. In this section, we discuss advanced analytical approaches to accurately utilize SFEN (Figure 5).

**Figure 5:**
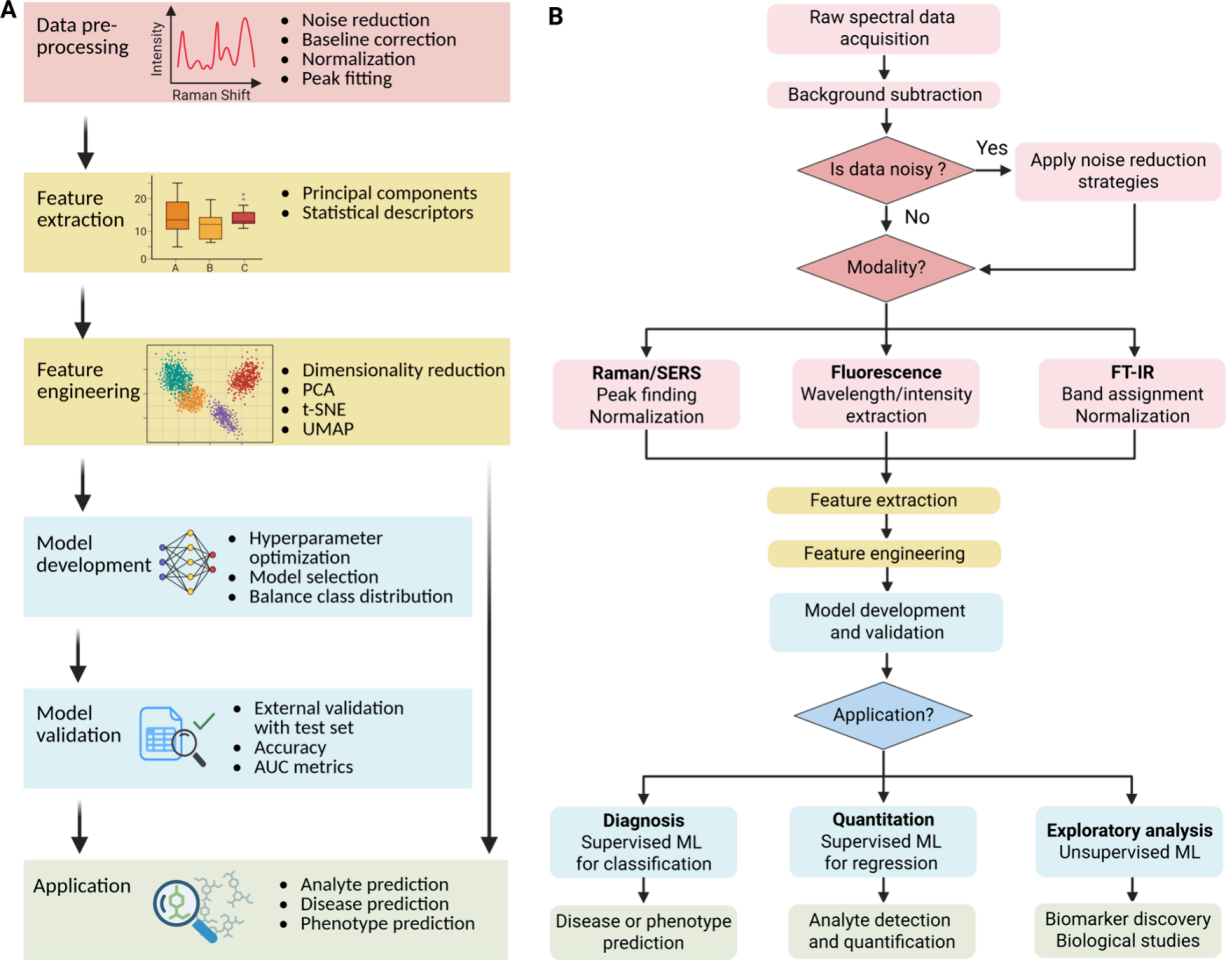
Machine-learning SFEN data set. (A) Workflow for SFEN data processing and ML integration. (B) Flowchart of the SFEN machine-learning pipeline across different modalities and applications.

#### 4.1.1 Data Preprocessing

Many data preprocessing techniques, such as noise reduction, baseline correction, and normalization, have been employed to refine SFEN signals. Savitzky–Golay filtering^97^ and Wiener estimation^98^ effectively reduce random noise in Raman spectra, increasing the signal-to-noise ratio. Savitzky–Golay filtering reduces noise through polynomial smoothing within a moving window, thus preserving spectral features,^99^ while Wiener filtering employs model-based estimation to minimize statistical error between noisy observations and ideal signals. Background interference, mainly arising from autofluorescence in tissue specimens, can be corrected by a pixel-by-pixel autofluorescence correction.^100–102^ Polynomial fitting and asymmetric least squares on spectral data to subtract baselines can mitigate unwanted scattering and/or substrate variability by correcting for background noise and visualizing underlying spectral features.^103–105^ To further ensure comparability between measurements and avoid systematic bias in multivariate analysis and machine learning, normalization strategies, including vector normalization, peak normalization, and total area normalization can be employed.^106–108^

#### 4.1.2 Feature Extraction

Feature extraction transforms processed spectral data into quantitative descriptors critical for classification, regression, or clustering tasks in SFEN. Universal features such as local maxima and their corresponding intensity or area under the curve can be systematically identified by algorithms that detect peaks above the background or fitted baselines. For increasingly complex data sets, for instance, hyperspectral imaging and multiplexed arrays, more sophisticated composite features are employed, including principal component scores,^109,110^ peak width, shape descriptors,^107,111^ knowledge distillation outputs,^112^ and custom statistical moments, such as skewness and kurtosis.^113^ These features help uncover subtle multivariate information often hidden in overlapping or convoluted spectral patterns.^108^ Furthermore, automated deep learning approaches enable hierarchical feature learning from large-scale or multiplexed biosensor data when the optimal descriptor is not known.

### 4.2 Feature Engineering and Visualization

Choosing the best feature extraction and visualization method depends on the spectral complexity of data and the main analytical objective. To enhance model generalizability, reduce the risk of overfitting, and improve the interpretability of the resulting prediction models, dimensionality reduction is essential. This includes excluding features with high correlation coefficients and adding features that significantly correlated with target classification. Composite and dimensionality reduction strategies excel in mixture discrimination and complex sample analysis, while automated feature learning is preferred for large-volume or discovery-oriented applications.

Exploratory data analysis employs dimensionality reduction techniques to elucidate separation between conditions, inform feature selection, and provide visual intuition. PCA linearly projects high-dimensional spectral data to orthogonal axes with maximum variance. It remains widely used both as a preliminary visualization and as an unsupervised feature reduction step prior to model training.^114,115^ PCA often reveals clustering that can anticipate diagnostic performance, although complex nonlinearities may go unnoticed. Calculating Pearson or Spearman correlation coefficients can also help identify and drop highly correlated features, preventing overfitting, even when individual features are statistically significant. An additional method for dimensionality reduction is t-distributed stochastic neighbor embedding (t-SNE), which is a nonlinear approach that preserves local structure,^116^ making it adept at visualizing subtle spectral distinctions, such as those between cell types or cancer subtypes.^117^ Uniform manifold approximation and projection are another emerging alternative to t-SNE, providing accurate representations of both global and local structure, and have shown promise in recent large-scale hyperspectral, single-cell fingerprinting applications, as well as single-cell DNA sequencing analysis.^118,119^ For array-based spectral sensors, visualization as heatmaps^120^ also aids in understanding the multidimensional response landscape and feature interdependencies.

### 4.3 Machine-Learning Models in Spectral Fingerprinting

#### 4.3.1 Unsupervised, Supervised, and Self-Supervised Methods

Unsupervised learning reveals innate groupings in high-dimensional data and is used for exploratory clustering, outlier detection, or stratification of unlabeled samples.^121^ Cluster analysis, hierarchical clustering, unsupervised feature selection, and dimensionality reduction methods such as PCA are typical tools used when unsupervised learning models.

Supervised learning predominates in diagnostic and biomarker detection settings, where class labels, such as disease state, health condition, cell type, etc., are known. Models learn to map extracted spectral features to labels, using algorithms such as support vector machines (SVM), random forests (RF), artificial neural networks (ANN), and, more recently, deep learning architectures (to be discussed in Sections 4.3.2 and 5.2.4).^122–126^ Prominent examples include early cancer detection,^127,128^ infectious pathogen identification,^129,130^ and macrophage phenotyping via NIR fluorescence,^41^ as discussed earlier.

A recent advancement in machine learning is the emergence of self-supervised models,^131^ which learn useful representations from large, unlabeled data sets by solving artificially constructed pretext tasks where the data itself serves as the source of supervision. Given that SFEN inherently produces large data sets with subtle or overlapping signal contrasts, self-supervised approaches may facilitate model pretraining, improve feature extraction, and support domain adaptation. For instance, models trained on data from clean, controlled systems can be adapted to complex biological environments or sensor performance can be validated across patient samples with diverse demographics in diagnostic applications. These capabilities contribute to the development of robust and scalable SFEN platforms for real-world deployment.

#### 4.3.2 Model Choice

The choice of model is critically shaped by the complexity of SFEN data and the trade-off between accuracy and interpretability. Interpretable, also known as white-box, models, such as *k*-nearest neighbors (KNN), decision trees, logistic regression, linear regression, and rule-based models, are particularly well-suited for tasks involving linearly varying relationships.^132,133^ These models are easily computed and allow direct insight into decision boundaries, making them transparent and straightforward for interpretation. On the other hand, more complex models, also known as black-box models, including neural networks (ANN), random forests (RF), and advanced ensemble algorithms like gradient boosting (XGBoost), excel at modeling nonlinear and highly interdependent spectral patterns.^134–136^ Support vector machines (SVM) are especially effective in high-dimensional feature spaces and with moderately sized data sets, particularly when using nonlinear kernels to delineate complex class boundaries. Random forests are widely used due to their robustness, resistance to overfitting, and ability to handle feature collinearity, with variable importance metrics supporting posthoc interpretability. Neural networks have recently been increasingly leveraged in biomedical applications, especially in pathology and imaging, where the data volume and complexity are high. Simple artificial neural networks work well for modestly sized feature arrays, while more advanced neural networks are particularly powerful for high-content spectral mapping and multiplexed sensor arrays.

It is important to note the inverse correlation between the model interpretability and accuracy. While white box models offer transparency and straightforward interpretation, they may be limited in capturing very complex relationships. Conversely, black-box models often achieve higher predictive accuracy, but at the expense of direct interpretability, necessitating post hoc explanation methods to better understand their decision-making processes.^137,138^ During model selection, it is important to balance the need for interpretability with the complexity of the classification task, optimizing for both performance and explainability to ensure robust, accurate, and trustworthy results in SFEN applications. For clinically relevant SFEN applications, models should be selected on not only performance but also amenability to explanation and alignment with known biology.

#### 4.3.3 Validation and Performance Metrics

Robust model validation and performance assessment are essential for translating SFEN results into clinically meaningful applications. Common practices include K-fold and nested cross-validation,^139^ which divide the data into multiple partitions to rigorously estimate predictive performance and minimize overfitting, a crucial consideration for small or imbalanced spectral data sets. Employing multiple train/test splits and repeated runs further ensures the stability and reproducibility of the results.

Gold standard metrics in diagnostic models include receiver operating characteristic (ROC) analysis and the corresponding area under the curve (AUC), effectively capturing the trade-offs between sensitivity and specificity. Confusion matrices provide additional clarity in prediction results by breaking down true positives, true negatives, false positives, and false negatives, enabling a detailed interpretation of classification errors and individual sample outcomes. External validation, e.g., multicenter validation, establishes generalizability and reliability across different instruments, laboratory protocols, and patient cohorts. By evaluating models on data sets acquired from diverse clinical centers, researchers can identify and mitigate center-specific biases, harmonize data preprocessing approaches, and boost confidence in real-world performance, which is a critical prerequisite for regulatory approval and clinical adoption.

When performance metrics are selected, the use case dictates which criteria are most critical. For screening and diagnostic applications, sensitivity at high specificity should be maximized since a low false-positive rate reduces unnecessary follow-up testing and patient anxiety. In prognostic or monitoring contexts, positive predictive value (PPV), negative predictive value (NPV), and calibration scores may take precedence, as correctly predicting outcomes or disease progression is paramount. For stratification or personalized medicine, balanced accuracy, sensitivity, and specificity (or F-score) are often emphasized to ensure robust, unbiased classification across multiple categories.

#### 4.3.4 Multimodal Data Integration

Integration of spectral fingerprinting with orthogonal data sources is a major and rapidly growing trend in the field of nanodiagnostics: Joint analysis of SERS and fluorescence images provides a multidimensional view of tissue, cell, or nanoparticle behavior.^140–143^ Combining spectroscopic data with electronic health records, genomic or proteomic markers, and patient metadata can enable holistic disease prediction and personalized diagnostics.^144–146^ The fusion of multimodal data often requires bespoke architecture, such as hybrids of CNNs and fully connected networks or advanced graph learning methods able to reconcile disparate data types and scales. Few studies have yet fully realized the clinical potential of such integration due to challenges in data harmonization, standardization, and regulatory oversight. There remains a vast, underexplored opportunity in developing open data sets, benchmarking challenges, and real-world deployment studies testing these integrative models in prospective trials for the advancement of precision biomedicine.

A critical enabler for SFEN translation and adoption will be the establishment of well-annotated spectral libraries that catalogue ENM-dependent and matrix-dependent fingerprints across biological contexts. Curated repositories of Raman, NIR fluorescence, and SERS spectra linked to sample type, nanomaterial formulation, acquisition conditions, and clinical labels would support benchmarking of preprocessing pipelines, facilitate the development of generalizable ML models, and accelerate mechanism-driven interpretation of new data sets. Open data initiatives modeled after existing consortia in genomics and imaging but tailored to spectral fingerprinting, inducing but not limited to raw data, meta data, and analysis code, would also promote reproducibility, reduce site specific biases, and lower the barrier for industry and regulatory bodies to independently evaluate SFEN performance. Public spectral libraries and harmonized data standards would further enable cross platform validation and transfer learning, allowing models trained on one instrument or cohort to be adapted to new devices, clinical sites, and disease indications.

#### 4.3.5 Interpretability and Explainability of ML for SFEN

For SFEN applications that target clinical decision making, model interpretability and explainability are as critical as the raw predictive power. While highly expressive architectures such as convolutional or attention-based neural networks can capture subtle, nonlinear multispectral signatures, their black-box nature can hinder regulatory acceptance and clinical trust, particularly when linking spectral features to known biological mechanisms. In this context, inherently interpretable models, like decision trees, remain valuable baselines, as they expose explicit relationships between spectral features and disease relevant states, enabling a more direct biochemical interpretation of SFEN readouts.

When more complex models are required to fully exploit SFENs high dimensional structure, a post hoc explainable ML tool can help reconcile performance with transparency. Feature attribution methods such as SHAP or integrated gradients, saliency maps for spectral or hyperspectral inputs, and layer wise relevance propagation can localize which wavelengths, bands, or sensor channels drive a given prediction, providing a bridge between model reasoning and underlying nanobiointeractions.^80,81^ In SFEN, these explanations can be mapped back onto specific vibration modes, fluorophore environments, or nanosensor chemistries, supporting hypothesis generation about mechanisms and facilitating cross-validation against orthogonal assays. Coupling these approaches with rigorous validation can improve transparency, credibility, and trust in SFEN based tools, while also guiding sensor design toward more mechanistically grounded, interpretable spectral fingerprints.

## 5 Future Directions and Challenges in Biomedicine

SFEN has transformed the landscape of biosensing by enabling ultrasensitive, multiplexed, and process-level detection of diverse biological phenomena. Through their unique optical and physicochemical properties, ranging from tunable fluorescence and plasmonic resonance to magnetic and vibrational signatures, SFEN approaches have been integrated into workflows spanning in vitro, ex vivo, and in vivo environments. The incorporation of advanced pattern recognition, particularly ML and artificial intelligence, has further enhanced their capacity for discerning complex biochemical signatures in health and disease. Key breakthroughs include robust discrimination of cell phenotypes and metabolic states at the single-cell level, multiplexed pathogen identification and disease staging using minimal sample volumes, and real-time tracking of nanomaterials and biomolecules in living tissues or plant systems. The convergence of SFEN technology with clinical and translational objectives positions these systems as highly effective for next-generation precision diagnostics and biomedicine applications (Figure 6).

**Figure 6:**
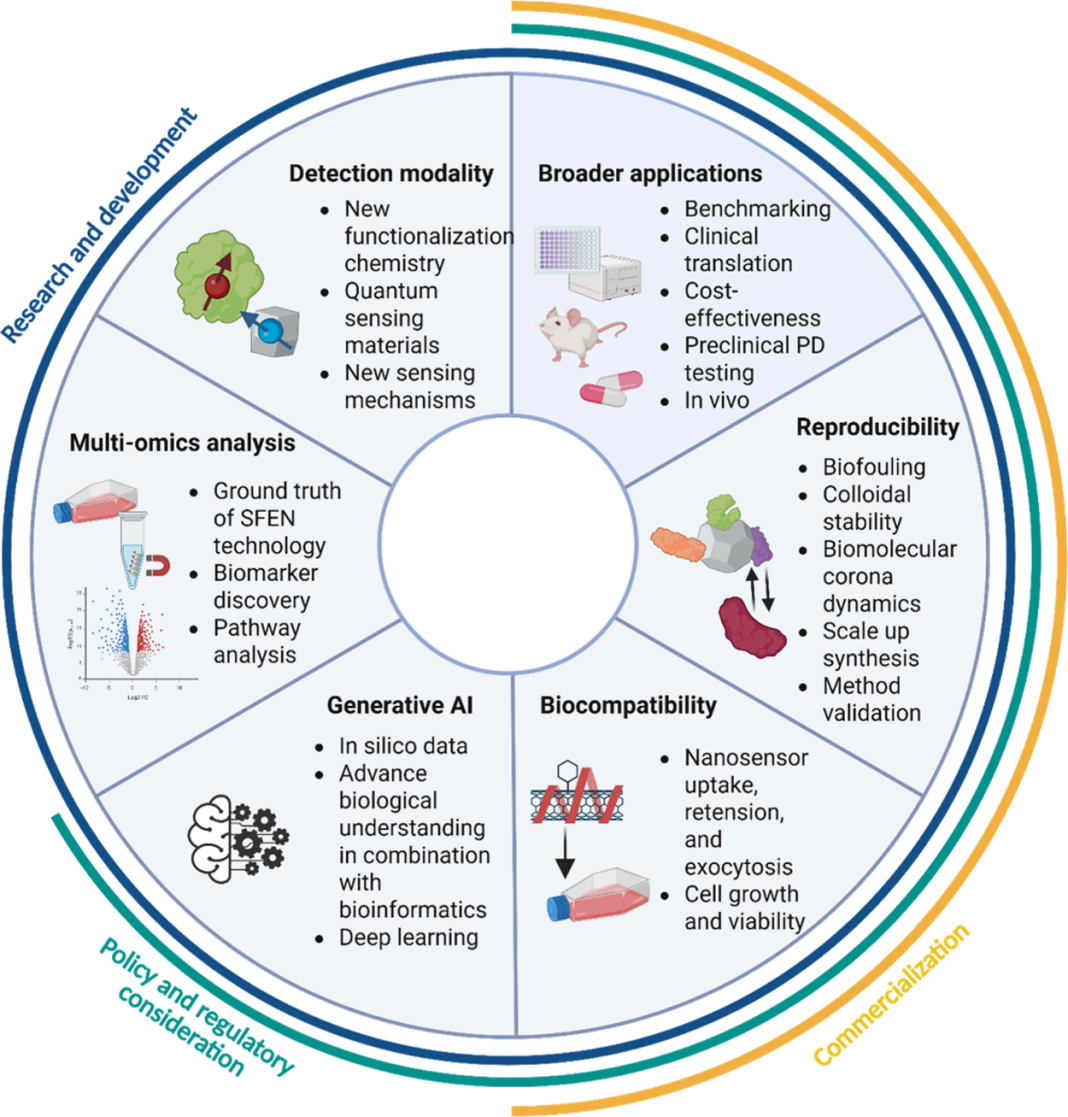
Integrated framework for advancing SFEN technologies across research, regulatory, and commercialization domains. The circular diagram highlights six key focus areas: (A) new detection modality, (B) broader applications, (C) reproducibility, (D) biocompatibility, (E) Generative AI, and (F) multiomics analysis. These are organized within three overarching domains—Research & Development (blue), Policy and Regulatory Consideration (green), and Commercialization (yellow) emphasizing the multidisciplinary efforts required for successful clinical translation and market adoption of nanotechnology-enabled biosensing platforms.

### 5.1 Challenges and Limitations

#### 5.1.1 Spectral Overlap and Reproducibility

Data reproducibility in SFEN technologies is often compromised by signal overlap in complex cellular environments such as tissue autofluorescence. This overlap makes multiplexed SFEN assays susceptible to crosstalk between different sensor signals, reducing specificity and complicating data interpretation. Avoiding tissue autofluorescence can largely circumvent issues arising from signal overlap. This can be effectively achieved by shifting SFEN readouts to the NIR spectral window, where autofluorescence is minimal and multiplexing is much more reliable.^131,148^ Beyond spectral tuning, several experimental and computational strategies can improve signal discrimination. Time resolved or lifetime-based detection methods separate fluorophores based on their distinct emission decay profiles, providing an orthogonal dimension to distinguish overlapping signals in crowded spectral spaces.^149,150^ Ratiometric sensing approaches, where emission intensities at two or more wavelengths are normalized, reduce the effects of intensity fluctuations and environmental variability on quantitative readouts.^151,152^ Furthermore, spectral unmixing algorithms such as non-negative factorization and principal component analysis-based approaches can deconvolve blended spectra to isolate pure component signals from mixed profiles in multiemitter complex systems.^153–155^ Employing algorithmic correction tools like the Huygens autofluorescence corrector can help estimate and subtract autofluorescence background,^156^ as well as cross-channel crosstalk computationally for persistent backgrounds.

#### 5.1.2 Biofouling and Matrix Effects

In realistic biomedical settings, SFEN readouts are strongly modulated by the surrounding biological matrix including proteins, lipids, metabolites, and extracellular vesicles present in serum, plasma, urine, saliva, or tissue homogenates. These components form dynamic coronas on engineered nanomaterials, alter the local refractive index, and introduce variable background signals that can shift or mask diagnostic spectral features, complicating analytical specificity and calibration. Matrix effects can also differ substantially between patient cohorts or collection protocols, challenging the transfer of models trained on clean or standardized samples to heterogeneous clinical samples.

As-produced engineered nanomaterials present a high energy surface that promotes the nonspecific binding of both charged and uncharged biomolecules in complex media, including macromolecules such as proteins and lipids as well as small molecules and ions.^157^ Engineered nanomaterials used in SFEN approaches are no exception. Collectively, this adhesion process forms a biomolecular corona and can “biofoul” a sensing surface, i.e., prevent the sensor from detecting a target analyte.^158,159^ It has been shown that the biomolecular corona can evolve over time.^160^ In a process known as the Vromen effect,^161^ less abundant but higher affinity molecules find the surface of the nanomaterial and displace lower affinity molecules. This known phenomenon presents a significant challenge for SFEN approaches due to temporal instability in the output data. While this biofouling process can unpredictably affect sensor performance over time, strategies have been developed in the nanomaterial sensing fields to limit the formation of a biomolecular corona, thus improving their functionality in complex biological environments.^158^ Many researchers employ PEG-based linear or branched polymer derivatives to fill empty regions of the nanosensors.^162^ When grafted to a nanomaterial at high density, this hydrophilic polymer limits nonspecific binding through strong steric repulsion forces. Zwitterions, in ligand or polymer form, are becoming increasingly popular as a strategy to limit biofouling.^163^ Their high hydration capability, due to the Coulombic forces between the zwitterions and water molecules, is stronger than hydrogen bonding and thus prevents proteins and other biomolecules from binding. In addition to PEG and simple zwitterionic strategies, other approaches are being developed, including dynamic hydrogels and surfaces, e.g., continuously degradable materials that limit adhesion of proteins and other biomolecules,^164^ as well as peptide amphiphiles^165^ and zwitterionic copolymer amphiphiles.^166^ Taken together, these strategies have significantly advanced the field of antibiofouling in recent years and have enabled SFEN technologies to function in complex biological environments.

#### 5.1.3 Other Environmental Effects

Conditions such as temperature, pH, ionic strength, and oxygenation further influence SFEN responses by modulating nanobio interactions, aggregation states, and intrinsic fluorescence or Raman scatter. Small drifts in excitation power, focus, or optical alignment across measurement sessions can also introduce systematic shifts, reducing longitudinal comparability and obscuring subtle biological changes. Addressing these issues requires the use of robust internal standards, radiometric or lifetime-based readouts, carefully controlled sampling workflows, and adaptive calibration or domain adaptation methods in downstream analysis.

#### 5.1.4 Scalability of Synthesis

Synthesizing engineered nanomaterials capable of spectral fingerprinting and transitioning SFEN technologies from a laboratory proof of concept to mass market adaption is a critical bottleneck. Unlike traditional chemical assays, SFEN relies on high-dimensional spectral fingerprints that are exquisitely sensitive to the physicochemical properties of ENMs. Batch-to-batch variations, such as slight deviations in the aspect ratio of plasmonic nanostructures or the chirality distribution of SWCNTs, can induce spectral shifts that misalign with pretrained machine-learning models. To mitigate this, the field must move beyond small scale batch synthesis toward a more continuous process like continuous flow microfluidic manufacturing,^167^ which may offer superior control over reaction kinetics and particle homogeneity. Furthermore, establishing rigorous quality control metrics and standardization of physical structure and characterization approaches^168^ is essential to ensure that a diagnostic model remains valid and performs well across different batches of produced sensors.

#### 5.1.5 Standardization and Regulatory Hurdles for Technology Translation

Successful commercialization and clinical translation require far more than moving laboratory prototypes to market. It demands an integrated life-cycle strategy, encompassing collaborative R&D, rigorous clinical validation, and a well-defined regulatory and market pathway.

The global regulatory landscape for nanomaterial-enabled biosensors remains fragmented. The FDA and other US regulatory authorities largely apply historical chemical regulations that inadequately reflect nanoscale attributes. This heterogeneity complicates international commercialization, as manufacturers must navigate inconsistent data requirements, test methods, and definitions.^168^ Efforts by the International Standards Organization to harmonize standards are ongoing, but slow. A universal regulatory code would accelerate the clinical translation and cross-border adoption of SFEN technologies.

For diagnostic applications of SFEN, rigorous benchmarking against clinical gold standards and stakeholder engagement is crucial to address regulatory hurdles. Benchmarking ensures that SFEN platforms meet the required performance metrics, such as low false-positive rates, high sensitivity at high specificity, and comparable performance in external multicenter validation relative to other clinical assays. Engaging stakeholders, including clinicians, regulators, and industry partners, will support transparent validation, regulatory confidence, and the successful adoption of SFEN-based diagnostics in healthcare settings.^168^

Another key hurdle is establishing a clear ground-truth for what this technology detects. Because SFEN signals often reflect complex, composite molecular fingerprints rather than a few biomarkers and often use poorly explainable machine-learning models, linking device output to established biological mechanisms is crucial. Employing interpretable or explainable ML frameworks, validating model rationale with biological evidence, and supporting transparency in decision-making are essential for technology validation.

### 5.2 Opportunities and Future Innovations

#### 5.2.1 Nanomaterials and Techniques That Can Expand SFEN Capabilities/Modalities

Current approaches in SFEN for precision biosensing are predominantly based on SERS and fluorescence modalities, employing a relatively narrow range of ENMs, such as gold and silver nanoparticles, quantum dots, and SWCNTs. While these platforms have demonstrated significant advances in sensitivity and multiplexing capability, the material choices and operational spectroscopies can be further expanded to capture the breadth of biological information.

Looking forward, there are major opportunities to expand SFEN capabilities by leveraging advances in quantum biology and the development of biocompatible and new quantum materials. Recent research is actively exploring materials such as chemically modified two-dimensional substrates like hexagonal boron nitride (hBN),^170^ quantum dots with engineered energy levels,^171^ and synthetic polymeric quantum materials that exhibit unique quantum properties, e.g., spin coherence and single-atom sensitivity.^172^ Quantum materials can transduce subtle biophysical and biochemical differences, such as electronic, nuclear spin, or vibrational changes arising from biological processes and signaling molecules, and manifest these as highly sensitive, label-free optical signatures.^173,174^ For instance, nitrogen vacancy centers in diamond have been used for magnetometry^175^ and quantum imaging to visualize biochemical processes without labels.^176^ Atomically thin hBN sensors with engineered spin defects can detect and control single nuclear or electronic spins at room temperature, potentially useful for next-generation bioimaging.^177,178^

Quantum biosensors integrating principles of superposition and entanglement can achieve ultrahigh sensitivity, rapid detection, real-time monitoring, and the simultaneous quantification of multiple targets, all in compact or even wearable formats. These developments are expected to expand the scope of SFEN beyond current SERS and fluorescence paradigms, opening the door to minimally invasive diagnostics, early disease detection, and dynamic cellular- or tissue-level phenotyping at high spatial and temporal resolution.

Beyond SERS and NIR, integrating SFEN with broadband vibrational spectroscopies such as FTIR^179–181^ and terahertz (THz) absorption^182–184^ presents an important future opportunity. These spectral regions capture collective vibrational, rotational, and hydrogen-bond modes that are highly sensitive to higher order structure, hydration, and long-range intermolecular interactions in proteins, lipids, nucleic acids, and supramolecular assemblies, coupling engineered nanomaterials with FTIR and THz modalities, for example, through nanostructured substrates, resonant metamaterials, or nanofluidic architectures, could amplify weak far IR/THz signals and translate subtle changes in macromolecular conformations, membrane organization, or phase behavior into robust, fingerprint like readouts. Such hybrid platforms would complement existing SFEN implementations by extending spectral coverage into regimes that report slower, mesoscale biophysical processes, thereby enriching the interpretation of complex biological states when integrated with ML and multimodal nano-omics.

#### 5.2.2 Integration with Nano-omics Approaches

Nano-omics, integrating nanomaterial-based proteomics, metabolomics, and genomics, represents a frontier for capturing molecular fingerprints with unmatched resolution. This approach has demonstrated utility in correlating circulating and tissue-based biomarker patterns, thereby uncovering mechanism-driven diagnostic and therapeutic targets.

Advanced spectral imaging techniques, when extrapolated to include ENM-enabled signal enhancement and targeting, have the potential to resolve dynamic biological processes within tissues or even single-cell events. SFEN technologies coupled with multimodal spatial omics can be used as nondestructive, real-time methods to infer gene-regulated biochemical changes and other insights into cellular function, without the need for sequencing. For instance, a recent study reported that the spatial metabolic profiles obtained from coherent Raman imaging were highly correlated with gene expression in *Caenorhabditis elegans* gonad.^185^ Nano-omics platforms also promise to accurately profile disease heterogeneity, track disease progression, and uncover mechanistic links among genotypes, phenotypes, and real-time biochemical states. Researchers have used gold nanoparticles to enrich and decode protein corona fingerprints that reflect serum proteome complexity, while others are exploring nanomaterials that respond optically or magnetically to metabolomic or nucleic acid content.^186^ Moving forward, high-dimensional nano-omics data from SFEN techniques will likely uncover new mechanistic disease biomarkers and pathways, supporting the next generation of precision, therapeutic tools.^300^

#### 5.2.3 Open Data Initiatives and Industrial Perspectives

A critical enabler for SFEN translation and adoption will be the establishment of well-annotated spectral libraries that catalogue ENM-dependent and matrix-dependent fingerprints across biological contexts. Curated repositories of Raman, NIR fluorescence, and SERS spectra linked to sample type, nanomaterial formulation, acquisition conditions, and clinical labels would support benchmarking of preprocessing pipelines, facilitate the development of generalizable ML models, and accelerate mechanism-driven interpretation of new data sets. Open data initiatives modeled after existing consortia in genomics and imaging, but tailored to spectral fingerprinting, inducing but not limited to, raw data, meta data, and analysis code, would also promote reproducibility, reduce site specific biases, and lower the barrier for industry and regulatory bodies to independently evaluate SFEN performance. Public spectral libraries and harmonized data standards would further enable cross platform validation and transfer learning, allowing models trained on one instrument or cohort to be adapted to new devices, clinical sites, and disease indications.

From an industrial standpoint, SFEN is well aligned with trends toward compact fiber-based or chip-integrated optical hardware and automated analysis pipelines that can be embedded in clinical laboratory instruments, point of care devices, or wearables. However, widespread adoption will require standardized manufacturing of ENMs with tightly controlled surface chemistry, robust quality control of spectral performance, and clear guidelines for long-term stability, safety, and regulatory classification of nanosensors. Partnerships between academic laboratories, instrument manufacturers, and clinical diagnostic companies could accelerate the codevelopment of SFEN hardware, reference materials, and software tools, while also informing regulatory frameworks for ML-assisted spectral diagnostics. Embedding SFEN readouts into existing clinical workflows, rather than positioning them as standalone technologies, may further lower integration barriers and highlight their value as a complementary, high frequency nano-omics layer for precision medicine.

#### 5.2.4 Generative AI and Deep Learning for Spectral Data Augmentation, Interpretation, and Model Validation

Deep learning architectures use neural networks with multiple layers to learn complex patterns from data and can process high-dimensional, multiplexed data. Deep learning has been widely adopted in image analysis applications, including digital pathology, medical imaging, and remote sensing, where large data volumes and high spatial heterogeneity are prevalent. Applying such models to SFEN will be beneficial for achieving substantial performance improvements as the scale and complexity of SFEN data sets continue to grow, especially when working with the hyperspectral imaging of tissues and multiplexed biosensor arrays.

Recent years have seen the emergence of deep neural networks, recurrent neural networks, and attention-based models (such as transformers), for spectral fingerprinting workflows in biosensing applications.^187–189^ Deep learning models excel at learning spatial and spectral features from hyperspectral images or large multiplexed array data,^120^ dramatically reducing the need for manual or engineered feature extraction. This approach enables automated identification of subtle spectral or spatial patterns that are difficult to capture with conventional descriptors. Transformers and attention-based models, although still emerging, hold strong promises for capturing long-range dependencies across spatial and spectral domains.

The adoption of generative AI and physics-informed models is revolutionizing spectral fingerprinting by enabling the creation of synthetic spectral data sets, which is especially advantageous when experimental data are limited or costly to obtain. Generative models, such as generative adversarial networks, variational autoencoders, and diffusion models, can be trained to produce high-fidelity, realistic spectral signatures that emulate the diversity and variance of experimental data.^190^ This capability supports robust data augmentation for model training, facilitating improved generalization, signal denoising, anomaly detection, and rare event modeling, all of which are crucial for sensitive biosensing and developing better diagnostic models.

Moreover, these generative models enable in-silico experiments under various physical and biological perturbations, including edge-case scenarios underrepresented in real data sets. In SFEN, where data sets have limited sample availability or substantial complexity in spectral features due to biological variability in the measurements, generative AI can augment data and introduce controlled perturbations. This enables deeper exploration of class boundaries, the interoperation of spectral fingerprints, and robust biomarker identification without increasing the laboratory burden and improving model performance and interpretability.

Integrating generative AI with advanced image analysis and computational microscopy brings further capabilities. For example, advanced imaging tools, such as broadband coherent Raman fingerprint imaging, high-speed coherent Raman imaging platforms, AI-augmented microscopy, and super-resolution imaging fusion, can integrate advanced denoising, segmentation, and data mining to produce dynamic, information-rich maps of biological tissues. This blend enables the simulation of whole-tissue and single-cell chemical homogeneity and interactively identifies rare or anomalous phenotypes from massive imaging data sets, ultimately expanding the interpretive and discovery power of spectral biosensing platforms. The use of conversational large language model-based agents, e.g., Omega,^191^ further automates image quantification, segmentation, and feature extraction, accommodating large, noisy, or heterogeneous spectral data sets with minimal manual intervention. These agents can interactively guide users in designing, testing, and interpreting complex spectral imaging workflows, automatically correcting errors, optimizing algorithms, and supporting reproducible science.

With the development of advanced models including deep learning and generative type AI models, the implementation of advanced validation strategies is critical not only to ensure the reliability of such AI-driven results but also to ensure the reliability of such biosensing systems. As a common practice, external validation using independent spectral data sets or cross instrument measurements can be utilized to assess AI model generalizability across different biological conditions or sensor platforms. Explainable AI frameworks, such as attention mapping, saliency analysis and feature attribution, can further provide interpretable insights into spectral features driving model decisions, bridging the gap between algorithmic outputs and biochemical relevance.^192^ In SFEN applications, such as those related to biomarker discovery, coupling deep learning predictions with experimental validation strategies, such as targeted biochemical assays or orthogonal spectroscopic measurements, strengthens the credibility of AI-driven biological targets and accelerates their translational potential in biosensing diagnostics.

## 6 Outlook and Conclusion

SFEN directly converts nanoscale physiochemical interactions into compact, information-rich spectral outputs that are complementary to conventional diagnostic technologies. SFEN can resolve subtle, multidimensional changes in fluorescence emission or Raman features that report on cell state, microenvironment, or bimolecular corona composition, enabling sensitive and quantitative discrimination of disease phenotypes at the point of care. Compared to omics platforms that profile thousands of molecules but require complex sample preparation, high costs, low throughput, and destructive nature, SFEN operates on minimally processed biofluids or live biological systems, can be implemented with fiber-based, wearable, or benchtop optical hardware, and enables minimally invasive monitoring of clinical states. Furthermore, SFEN leverages engineered nanomaterials as both transducers and nanoscale integrators of local biochemical complexity, amplifying low abundance molecular signatures into robust optical fingerprints and facilitating biomarker discovery process.^193–195^

Despite these advantages, several key questions must be addressed before SFEN can be broadly deployed in routine clinical practice. Some unresolved challenges include establishing mechanistic ground truth for SFEN readouts in complex biological matrices, defining how nanobiointeractions and biomolecular corona dynamics shape disease-specific spectral fingerprints across diverse patient populations, and determining the extent to which models trained on curated data sets generalize to real-world, longitudinal use. Further work is also needed to standardize acquisition protocols and spectral libraries across instruments and sites, develop robust, interpretable ML frameworks that link spectral features to known biochemical pathways, and systematically evaluate the safety, biocompatibility, and long-term performance of ENM-based sensors in vivo. Addressing these open questions through coordinated efforts in materials design, clinical study design, open data sharing, and regulatory science will be essential to fully realize SFEN as a reliable platform for precision biosensing and diagnostics.
